# Spatio-Temporal Statistical Characterization of Boundary Kinematic Phenomena of Triaxial Sand Specimens

**DOI:** 10.3390/ma15062189

**Published:** 2022-03-16

**Authors:** Yichuan Zhu, Zenon Medina-Cetina, Alma Rosa Pineda-Contreras

**Affiliations:** 1 Civil & Environmental Engineering Department, Temple University, Philadelphia, PA 19122, USA; yichuan.zhu@temple.edu; 2 Zachry Department of Civil & Environmental Engineering, Texas A&M University, College Station, TX 77843, USA; 3 Laboratorio de Geoinformática, Instituto de Ingeniería, Universidad Nacional Autónoma de México UNAM, Torre de Ingeniería 2° Piso, Circuito Escolar C.U., Coyoacan, Mexico City 04510, Mexico; apinedac@iingen.unam.mx

**Keywords:** statistical analysis, 3D-DIC, spatio-temporal process, localization effects, triaxial compression test

## Abstract

This paper follows up on a reference paper that inspired MDPI’s Topic “Stochastic Geomechanics: From Experimentation to Forward Modeling”, where global and local deformation effects on sand specimens are fully described from high resolution boundary displacement fields, and supported by its experimental database, which is open to the scientific community for further study. This paper introduces the use of spatio-temporal statistics from a subset of such an experimental database to characterize the specimens’ spatio-temporal displacement fields, populated by repeating a set of triaxial compression tests on drained, dry, vacuum-consolidated sand specimens, tested under similar experimentally controlled conditions. A three-dimensional digital image correlation (3D-DIC) technique was used to measure the specimens’ boundary displacement fields throughout the course of shearing under axial compression. Spatio-temporal first- and second-order statistics were computed for different data dimensionality conditions (0D, 0D-T, 1D-T, 3D-T) to identify and characterize the dominant failure mechanisms across different testing specimens. This allowed us to quantify localization phenomena’s spatio-temporal uncertainty. Results show that the uncertainty captured along the deformation process across different dimensionality conditions can be directly associated with different failure mechanisms, including localization patterns, such as the onset and evolution of shear, compression, and expansion bands. These spatio-temporal observations show the dependencies between locally distinctive displacement regions over a specimen’s surface, and across different times during a specimen’s shearing process. Results of this work provide boundary spatio-temporal statistics of experimental evidence in sands, which sets the basis for the development of research on the numerical simulation of sand’s constitutive behavior. Moreover, it allows to add a new understanding on the effect of uncertainty on the mechanistic interpretation of sands’ kinematic phenomena.

## 1. Introduction

Soils in their natural environment have an inherent variability associated with their geologic origin observed through their specific physical and mechanical properties, and their stratigraphic spatial distribution. These are associated with a wide range of material properties. Soils’ variability in particular represents a unique random (space) and stochastic (time, or space and time) geoscientific and geoengineering challenge, starting with the quantification of such variability into a metric of uncertainty. For instance, in typical laboratory triaxial compression tests of soil specimens, various mechanistic failure modes can be observed among soils with similar physical characteristics, even if the experimental process is conducted under similar controlled conditions [1,2]. The observed variability in standardized soil tests may be the result of inherent heterogeneity of material, variations associated with testing apparatus, human-introduced errors, or a combination of these. Assessing the effect of randomness of soils’ mechanical properties is a challenge, particularly when attempting to identify and characterize their underlying failure mechanisms. A known limitation to assessing this effect is the cost associated with testing and the limited knowledge of the effect of geomaterial experimental randomness. For a systematic characterization of a soil’s mechanical failure process, a statistical description is thus not only desirable but a necessary complement to conventional laboratory tests, used to define design parameters, and to investigate the dominant failure mechanisms, including the uncertainty associated with them when these evolve in space and time.

Soils’ continuum failure begins with local material anomalies as fine as grain scale [3,4]. However, in traditional plane or triaxial compression tests, for instance, materials are collectively considered “homogeneous”, because failure phenomena between points of measurement (i.e., load cell and displacement transducer) are in fact averaged material responses, which produce a “global” identification and characterization of mechanical failure. To properly characterize the heterogeneous deformation mechanisms of a granular material, full-field measurements of testing specimens are necessary. The first experimental measurements of this type can be traced back to as early as the 1960s, when Roscoe [5] used a 150 kV X-ray apparatus to check the nonuniform behavior of specimens. In the 1980s, Desrues et al. [6,7] used X-ray tomography to investigate strain localization patterns in sand, including orientation, thickness, and volumetric behavior. Microstructure and evolving mechanisms inside of the shear band have been observed as well using a microfocus X-ray CT system [8,9,10]. These studies provided a valuable insight into particle interaction and density variations within a specimen. Nevertheless, limitations in data acquisition resources have led to most analysis being conducted post-mortem or by capturing data over wide strain increments; this procedure could lead to misidentification of chronologically occurring localized strains over each short time period, perhaps resulting in strain localizations appearing simultaneously in all regions, as noted by Desrues and Viggiani [1]. One significant improvement to overcome this issue is to perform ‘in-situ’ X-ray scanning during the course of loading [7,10]. More recent studies have even incorporated particle identification and tracking algorithms to assess the link between grain morphology and localization effects [11,12,13].

Over the last two decades, the 3D digital image correlation (3D-DIC) technique coupled with experimental testing of geomaterials has proved to be an effective method to quantify high-resolution (space), high-frequency (time) grain-scale displacement fields in a spatio-temporal continuum representation of the specimen’s boundary. The result of 3D-DIC analysis shows micro- to meso-scale boundary displacement fields, which allows for the identification of deformation characteristics of particle to groups of soil particles. The relatively convenient implementation and nearly spatio-temporal continuous description of the kinematics of this technique make it increasingly popular for studying geomaterial mechanistic failure mechanisms [14,15,16]. For example, Rechenmacher [17] used DIC to quantify the triggering of the formation of persistent local effects such as shear bands in 2D, and to investigate kinematic properties within shear bands of sand specimens undergoing plane strain deformation. Furthermore, Rechenmacher et al. [3,18,19] evaluated shear, rotational, and volumetric strains; build-up and collapse of force chains; and vortex structures on a series of deformed soil samples, in a spatio-temporal manner (2D-T).

In spite of rapid proliferation of experimental techniques and processing methods to account for boundary and full-body continuum material characterization, proven research technologies have not yet been standardized. At the field scale, spatial variability of soil properties has been modeled by the use of random field theory through the scale of fluctuation [20,21]. However, at the laboratory specimen scale, material heterogeneity is not commonly accounted for to calibrate constitutive models and to better reproduce failure mechanisms. It is accounted for even less often to systematically assimilate a statistical characterization of soil spatio-temporal deformation patterns after testing the same geomaterial under the same experimental conditions, to better predict “not-sampled but likely” failure mechanisms.

A focus on local material behavior is an inherent acknowledgment of random or heterogenous effects, which naturally lead to variable failure mechanisms, contrary to the homogeneous geomaterial assumption made for most constitutive models. Additionally, a good example of investigation should follow a multi-level inspection of a deformation process across different scales [22], which indicates that multi-scale statistical investigation can help improve our current understanding of failure mechanisms of soil across different spatio-temporal scales. We hypothesize that such an approach will contribute to a better definition of soil constitutive models and a better assessment of the impact of soils’ uncertainty characterization on geoscientific and geoengineering processes and structural designs.

This paper introduces a subset from a comprehensive database that provides evidence about the effect of localization on sands’ constitutive behavior, derived from the use of the 3D-DIC technique [2,23,24]. In Section 2, we present the data sampling process: a data ensemble of spatio-temporal displacement fields populated from repeating a set of triaxial compression tests of drained, dry, vacuum-consolidated heterogenous sand specimens tested under similar experimentally controlled conditions. In Section 3, the statistical characterizations are presented in different dimensionality modes following an inductive approach: (a) the 0D-0T data ensemble represents the sand tests’ “global” mechanical properties, (b) the 0D-T data ensemble represents the sand tests’ axial stress–strain and the axial strain–volumetric strain curves, (c) the 1D-T data ensemble represents the sand tests’ boundary vertical and radial displacement curves, and (d) the 3D-T data ensemble represents the sands tests’ boundary 3D displacement fields’ surfaces.

For each dimensionality condition defined above, a standard descriptive-statistics analysis was formulated, consisting of first- (mean and standard deviation analysis) and second-order (correlation structural analysis) statistics, as applicable to that condition’s spatio-temporal nature. The purpose of these statistics is to produce a new set of inferences focused on failure mechanisms and their corresponding local effects as they evolve in space and time. The results provide unique statistical insights into spatio-temporal displacement fields and localization effects for the same reconstituted sand, tested under the same laboratory-controlled triaxial compression conditions. When this new set of inferences (1D-T and 3D-T) is added to the standard interpretation of triaxial tests (0D-0T and 0D-T), a new set of interpretations and knowledge is produced that can serve as the basis for developing statistical “virtual” simulations statistically consistent with the “real” laboratory experimental data, which can further reveal the impact of material heterogeneity on soils’ constitutive behaviors.

## 2. Soil Experiments

### 2.1. Triaxial Compression Test

The data ensemble introduced in this work is a subset of an experimental database that includes a series of drained, vacuum-consolidated triaxial compression tests made fully available in a preceding paper by Medina-Cetina et al. [24]. Included are tests using construction sand graded as SP, selected to reconstitute sand specimens because it had a color spectrum appropriate for pattern recognition during DIC analysis. Table 1 gives sample characteristics of 17 nominally similar tests in terms of aspect ratio, initial density, relative density, friction angle, and stress ratio at peak state. Most specimens were constituted through vibratory compaction in three uniformly compacting layers, and four were prepared using dry pluviation with controlled drop-height to reach a similar initial density.

**Figure 1:**
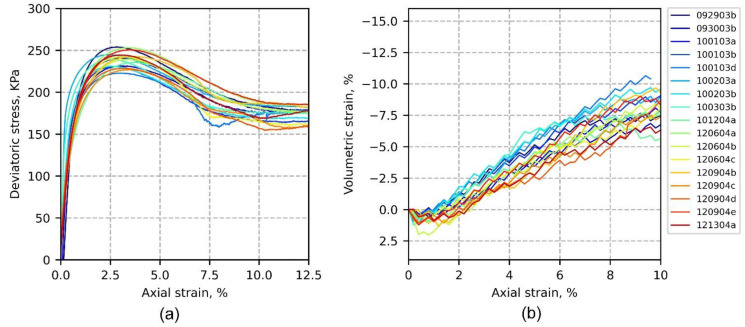
Triaxial stress–strain curves of the 17-test ensemble (**a**) and axial strain–volumetric strain curves of the 17-test ensemble (**b**).

The triaxial frame setting was similar to that of the conventional system except that the Plexiglas cell was removed to avoid light reflection during stereo-image capture of the shearing. The testing layout was presented in Figure 1 of the paper by Medina-Cetina et al. [24]. All specimens were vacuum consolidated at 40 KPa and compressed under a strain control rate of 0.2%/min. Figure 1 presents the global stress–strain and volumetric strain responses for the 17 tests considered for this paper’s ensemble. It shows how variability grows as soon as the compression starts, indicating a heteroscedastic behavior up to the stress peak. In the post-peak regime, data variability seems homoscedastic, in general (Figure 1a), although with some continued scattering for the volumetric strain (Figure 1b). Because of the triaxial compression setting used for testing, the volumetric strain curves were computed based on 3D-DIC boundary measurements. Details about the method used to compute these curves are discussed in [2,24]. Further investigation of the specimen’s boundary local effects associated with likely failure mechanisms would require a high-resolution description of the full-field spatio-temporal displacement fields, along with its spatio-temporal statistical analysis. This type of analysis is presented in following sections, from the lowest to the highest dimensionality possible, allowing the relationship between the variability of the material response and the observed boundary local deformation effects to be explored.

### 2.2. 3D-DIC

This work is based on the use of the 3D-DIC technique to measure high-resolution boundary displacement fields during the shearing of all sand specimens. The 3D imaging system consisted of two digital cameras set up in front of a soil specimen that had undergone a triaxial compression test. During the process of shearing, these two cameras took synchronous images every 15 s (0.05% of axial strain), producing a set of stereo digitized images using the software VIC-Snap by Correlated Solutions Inc. (Irmo, SC, USA) [25]. To assimilate graphical information into full-field displacements, subsets of pixels between two digital images were identified and correlated through the DIC algorithm to produce high-resolution spatio-temporal displacement fields [26]. The resulting dataset after this step is a set of incremental (Eulerian) DIC displacement data, which had to be “pieced together” [2,24] to produce displacement fields covering approximately one-fourth of the cylindrical sector.

**Figure 2:**
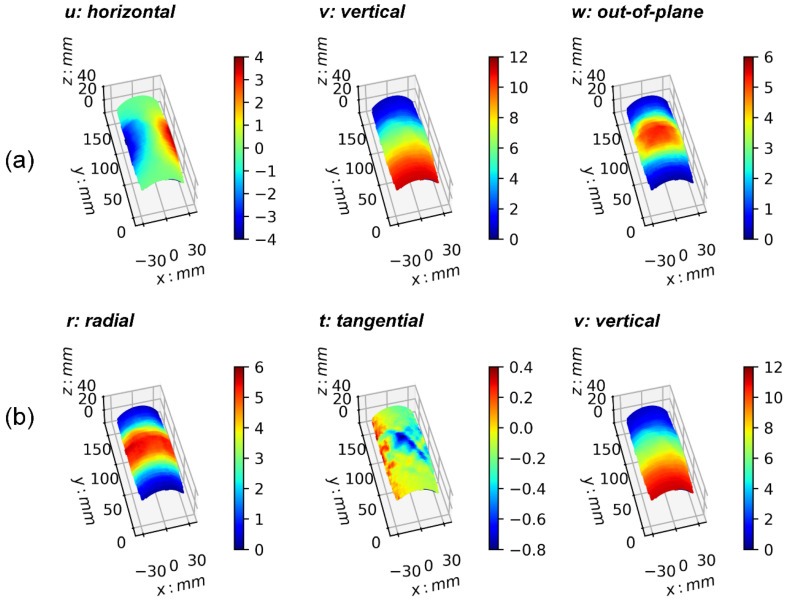
Example of 3D displacement field. Test 092903b at 7% of axial strain. (**a**) Displacement field projections of Cartesian components in horizontal, vertical, and out-of-plane directions (left to right). (**b**) Displacement field projections of cylindrical components in radial, tangential, and axial directions.

Figure 2 is an example of 3D-DIC displacement fields superimposed over a specimen’s deformed boundary shape (test 092903b) at 7% of axial strain. The first row (a) is a “snapshot” of the spatio-temporal 3D displacement fields under the Cartesian coordinate system. Displacement fields, from left to right, are *u*, *v*, and *w* (i.e., projection of Cartesian vector components, in millimeters), which represent local displacements in the horizontal, vertical, and out-of-plane directions, respectively. Displacement component *u* shows negative and positive displacements, indicating horizontal motion in the left and right directions. Displacement component *v* shows vertical motion of the specimen, which is evident at the bottom because of the upward loading method, and close to zero displacements at the top because of the fixed boundary at the specimen’s top. Displacement component *w* shows significant out-of-plane motion at the middle of the specimen, which can be associated with the specimen’s bulging effects during shearing. The second row (b) of Figure 2 is a similar “snapshot” of the 3D displacement field as in the first row (a) but using cylindrical coordinates. Displacement components *r*, *t*, and *v* represent, from left to right, projection of the displacement vector along radial, tangential, and vertical directions (i.e., projection of cylindrical vector components, in millimeters), respectively. Radial displacements are positive, representing the specimen’s radial expansion, which is maximum in the middle and close to zero at the bottom and top of the specimen because of the friction effect at each end. The tangential displacement field ranges from negative to positive, representing the displacement component tangential to the boundary of the specimen. The vertical displacement component shows positive displacements only, as in the same projection of vector component *v* in the Cartesian representation (middle figure on row a).

## 3. Statistical Characterization of Spatio-Temporal Boundary Displacement Fields

### 3.1. “0D-0T” Data Ensemble

**Figure 3:**
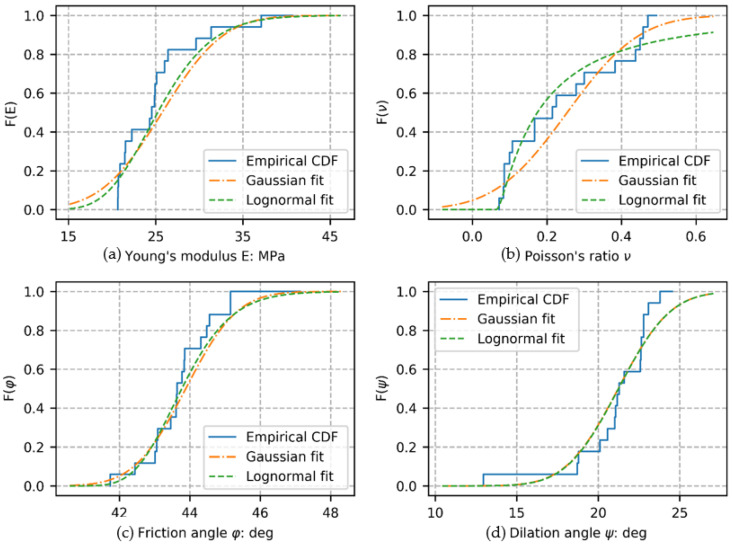
Empirical cumulative density function of each constitutive parameter, including Gaussian and lognormal model fits (as descriptive reference only): Young’s modulus (**a**), Poisson’s ratio (**b**), Friction angle (**c**), and dilation angle (**d**).

This section introduces a summary of the statistical characterization of the main mechanical parameters computed from the material stress–strain and volumetric strain responses. Conventional constitutive parameters, including Young’s modulus E, Poisson’s ratio ν, friction angle φ, and dilation angle ψ, were estimated for each specimen from the global stress–strain and volumetric strain curves presented in Figure 1. Table 2 is a summary of these parameters’ first-order statistics (there is no characterization of space or time in these parameters). Figure 3 presents the empirical cumulative density function (eCDF) of each of these parameters, together with Gaussian and lognormal fitting curves (using statistics from Table 2), for descriptive comparison purposes only (probabilistic modeling of each parameter is out of the scope of this paper).

### 3.2. “0D-T” Data Ensemble

**Figure 4:**
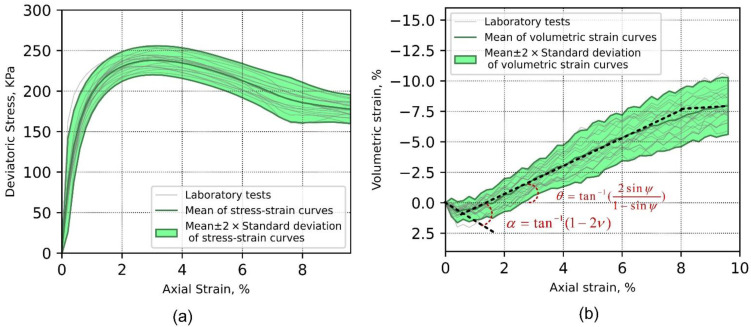
First-order statistics of axial stress–strain curves (**a**) and axial strain–volumetric strain curves (**b**).

The “0D-T” data ensemble represents the axial stress–strain and axial strain–volumetric strain curves ensemble shown in Figure 1, from which the first-order statistics (mean and standard deviation) were calculated. First-order statistics of the global deviatoric stress and volumetric strain as a function of axial strain for all tests are depicted in Figure 4a,b, respectively. The axial strain ranges from 0 to 9.6%, which was determined according to the extent of sampled DIC data available for all tests. Figure 4a shows the averaged stress response reaches its peak at close to 3.2% of the axial strain, with mean and standard deviation equal to 237.89 and 8.92 kPa, respectively. After the peak stress, the softening of stress–strain material responses shows a nominally constant variation (homoscedastic) through the last shearing stage (axial strain of 9.6%). Figure 4b shows that volumetric dilation starts developing during the elastic phase. Intersection of the compression and dilation linear trends happens before 1% of the axial strain, whereas the critical state seems to be reached around 8% of the axial strain, as indicated by the intersection of the linear fits. Variability of the volumetric strain seems to grow as the test progresses, as indicated by the standard deviation band plotted around the mean (heteroscedastic); it is hypothesized that variability is associated with the development of competing triggering failure mechanisms, as discussed in subsequent sections.

In addition to mean and standard deviation, correlation analysis (second-order statistics) was performed on the same data ensembles (axial stress–strain and axial strain–volumetric strain curves) in order to characterize the correlation structure of global material responses. It was proposed to use the classical linear Pearson correlation coefficient to estimate the degree of association between vectors of deviatoric stresses or volumetric strains at different loading levels (i.e., axial strains). To calculate the correlation coefficient, for instance, of any given two random variables—$S_{i}\left(\varepsilon_{a,i}\right)$ and $S_{j}\left(\varepsilon_{a,j}\right)$—which represent two deviatoric stress vectors defined at axial strain levels $\varepsilon_{a,i}$ and $\varepsilon_{a,j}$, respectively, the covariance between $S_{i}$ and $S_{j}$ can be defined as:

(1) $$\mathrm{cov}(S_{i},S_{j})=E[(S_{i}-E(S_{i})(S_{j}-E(S_{j})]$$

The covariance can be normalized to produce the correlation coefficient,

(2) $$\rho (S_{i},S_{j})=\frac{\mathrm{cov}(S_{i},S_{j})}{\sigma (S_{i})\sigma (S_{j})}$$

where $\sigma \left(S_{i}\right)$ and $\sigma \left(S_{j}\right)$ represent the standard deviation of deviatoric stress data from random variables $S_{i}$ and $S_{j}$, respectively.

**Figure 5:**
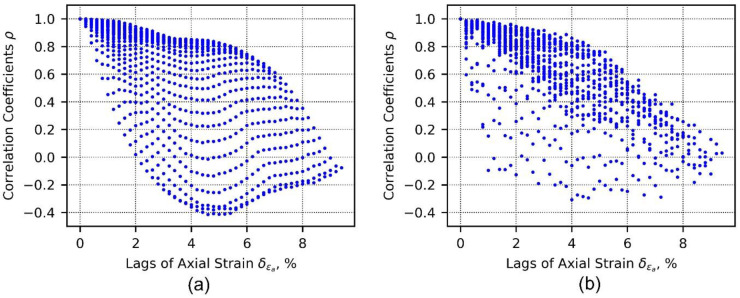
Correlation coefficients computed from axial stress–strain data ensemble (**a**) and axial strain–volumetric strain data ensemble (**b**).

Figure 5 shows correlation coefficients computed from the axial stress–strain data ensemble and axial strain–volumetric strain data ensemble, respectively. X axes in Figure 5 represent lags of axial strain, $\delta_{\varepsilon_{a}}$. When $\delta_{\varepsilon_{a}}=0$, it corresponds to auto-correlations of vectors of data corresponding to 48 different axial strain levels (i.e., from 0.2 to 9.6% with an increment step of 0.2); thus, 48 correlation coefficients are calculated. On the other hand, when $\delta_{\varepsilon_{a}}=9.4$, only one correlation coefficient can be calculated that corresponds to vectors of data corresponding to axial strain at 0.2 and 9.6%, respectively.

### 3.3. “1D-T” Data Ensemble

#### 3.3.1. “1D-T” Vertical Displacement Field

**Figure 6:**
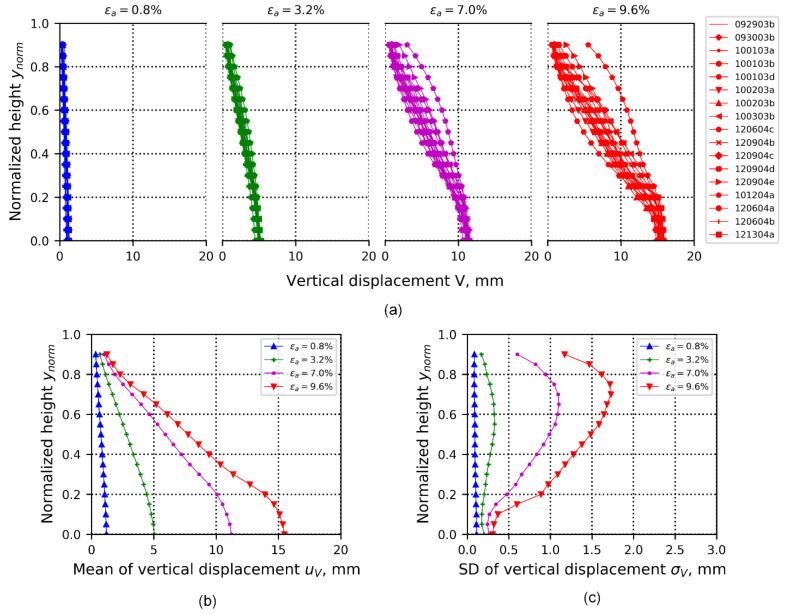
(**a**) Averaged vertical data ensembles calculated from 17 tests at four loading stages—0.8%, 3.2%, 7.0%, and 9.6% of axial strain—for 1D-T data ensembles. The vertical displacement at each specimen height is estimated through averaged vertical displacements that are captured by 3D-DIC. (**b**) Mean profiles of data ensembles of averaged vertical displacements shown in (**a**). (**c**) Standard-deviation profiles of data ensembles of averaged vertical displacements shown in (**a**).

The first dimensional spatio-temporal representation of the 3D-DIC data ensemble is its 1D-T data ensemble representation. This ensemble consists of the averaged vertical and radial displacement fields at the same height of the specimen, and across the vertical profile of each specimen. This averaged representation is produced in order to better interpret the occurrence of local deformation effects on the specimen’s boundary, as if data could be collected in 1D-T only. Vertical profiles presented here represent data interpolated from each test taken at every 0.04 of normalized specimen height. At the top of the specimen, data are incomplete because retrieving data is difficult close to the boundary of the sand specimen and the porous stone for some tests, which defined the maximum height of data available for the full ensemble (maximum height for which data are available for all of the data ensemble). The range of axial strain for this analysis is 0.0 to 9.6%, with 0.2% (1 min) incremental steps. Figure 6a presents 1D averaged vertical-displacement data ensemble profiles, where the specimen height was normalized based on initial sample geometry, at four loading stages: 0.8%, 3.2%, 7.0%, and 9.6% of axial strain (representing compression stages of elastic, peak, softening, and critical state, respectively). Figure 6b,c present the spatio-temporal first-order statistics of datasets introduced in Figure 6a. These figures show that after the peak stress ($\varepsilon_{a}=3.2\%$), the bottom part, representing nearly 20% of specimen height, exhibits approximately homogeneous upward displacement (Figure 6b). This homogeneous deformation can be associated with relatively higher density at the lower part of the specimen (observed particularly in samples prepared using the vibratory-compaction method). Above the normalized height of 0.2, deformation patterns show a linear trend, which is gradually reduced to zero at the top of the specimen (see axial strain profiles at 7.0 and 9.6% of axial strain). The profile of the standard deviation of the averaged vertical displacement is presented in Figure 6c, and shows how randomness of the averaged vertical displacement is nonuniform and reaches its peak consistently at $y_{norm}=0.75$, indicating a perturbation that can be associated with a local deformation effect (e.g., shear or compression band). All testing specimens were fixed at the top and loaded from the bottom with the same loading rate, which explains the consistent low uncertainty at zero height.

To calculate the cross-correlation for the 1D-T data ensemble, for instance, we used two random variables, $V_{i}\left(y_{nrom,i},t_{i}\right)$ and $V_{j}\left(y_{nrom,j},t_{j}\right)$, representing two 1D-T displacement vectors defined at normalized locations $y_{nrom,i}$ and $y_{nrom,j}$ and specific loading times $t_{i}$ and $t_{j}$, respectively. The covariance given to $V_{i}$ and $V_{j}$ can be defined as:

(3) $$\mathrm{cov}(V_{i},V_{j})=E[(V_{i}-E(V_{i})(V_{j}-E(V_{j})]$$

The covariance can be normalized to produce the correlation coefficient:

(4) $$\rho (V_{i},V_{j})=\frac{\mathrm{cov}(V_{i},V_{j})}{\sigma (V_{i})\sigma (V_{j})}$$

where $\sigma \left(V_{i}\right)$ and $\sigma \left(V_{j}\right)$ represent the standard deviation of displacement data from random variables $V_{i}$ and $V_{j}$, respectively.

**Figure 7:**
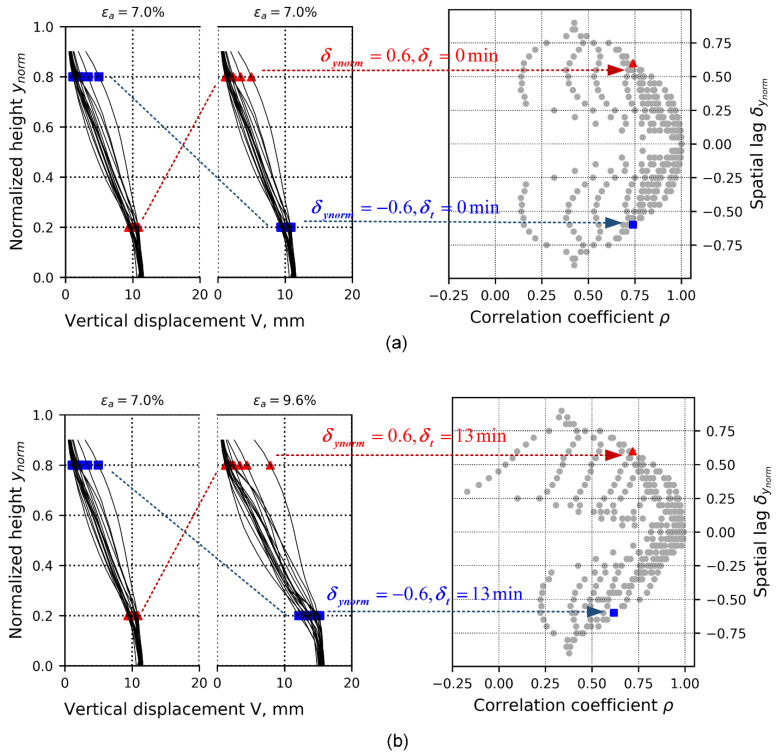
Cases illustrating computing correlation coefficients toward the 1D-T vertical displacement field. (**a**) Calculation of auto-correlations of data ensemble at loading stage of 7.0% of axial strain. Red triangles represent the computing case that has positive spatial lag, and blue squares represent the computing case that has negative spatial lag. The resulting correlation coefficients are plotted against spatial lags along the vertical profile of the specimen. (**b**) Calculation of cross-correlations of data ensembles at loading stages of 7.0 and 9.6% of axial strain. The procedure is similar to that of Figure 6a, except that time lag, $\delta_{t}$, is non-zero and needs to be interpreted from two loading stages.

Figure 7a,b show two cases to illustrate the computation of the auto- and cross-correlation analyses. Auto-correlation denotes correlation only within the same data ensemble at a given time—namely, time lag $\delta_{t}=0\;\min$—whereas cross-correlation denotes correlation computed between data ensembles at different times (i.e., loading stages)—namely, time lags $\delta_{t}\neq 0\;\min$. Figure 7 presents both auto- and cross-correlation cases for the 1D-T data ensemble. Figure 7a presents the computation of two correlation datasets distanced $\delta_{y_{norm}}$ located at normalized heights of 0.2 and 0.8 at 7.0% of the axial strain. Since $\delta_{ynorm}$ can be measured in either direction depending on which vector is defined as $V_{i}\left(y_{nrom,i},t_{i}\right)$ and $V_{j}\left(y_{nrom,j},t_{j}\right)$ (along the normalized height), thus $\delta_{ynorm}$ can have both positive and negative values. For the auto-correlation case, as highlighted by the squared and triangle symbols located in the positive and negative sides of $\delta_{y_{norm}}$ in Figure 6a, the correlation structure is symmetrical with respect to the horizontal line $\delta_{ynorm}=0$. In the case of cross-correlation (between data ensembles at 7.0% and 9.6% of axial strain), the correlation structure is not symmetric (Figure 7b).

**Figure 8:**
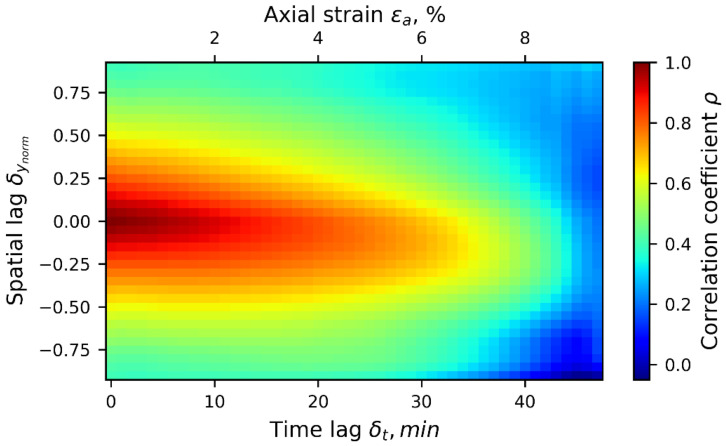
Smooth hypersurface representing the spatio-temporal empirical correlation structure for the 1D-T data ensemble of averaged vertical displacements.

To better represent the second-order statistics of the 1D-T data ensemble, all 48 stages of correlation fields were calculated, from strain levels 0.0 to 9.6%, including both auto- and cross-correlations. The result is a cloud of correlation points defined in spatial lags, $\delta_{y_{norm}}$, ranging between −1 and 1, and in time lags, $\delta_{t}$, ranging from 0 to 48 min (only positive temporal lag is considered due to correlation symmetry). This representation of the general trend of the correlation structure of the 1D-T data ensemble is shown in Figure 8, which shows the projections of a cubic spline interpolation curve fitting the cloud of points describing the spatio-temporal correlation structure of the displacement field, where the horizontal and vertical axes represent the temporal and spatial lags, respectively, and the colour bar indicates the value of the correlation coefficient, $\rho \left(\delta_{y_{norm}},\delta_{t}\right)$. The correlation plot follows the approximate symmetrical shape with respect to the axis $\delta_{y_{norm}}=0$, following the description of the computation of ρ shown in Figure 7. There is some skew beyond the $\delta_{t}$= 10 min in the time-lag domain (equivalent to $\Delta \varepsilon_{a}=2\%$). Correlation values higher than, for example, ρ>|±0.75| extend to a vertical lag distance of $\delta_{y_{norm}}<\left|\pm 0.33\right|$ and to a time lag of $\delta_{t}<\left|\pm 20\right|$ (time-lag equivalent to ±2.0% of axial strain). Beyond this region, the influence of local data drops in space and time because of randomness effects associated with the development of local deformation phenomena.

This set of results indicates that localization effects along the specimen’s boundary surface cannot be fully characterized by the interpretation of the first-order statistics (Figure 6). As previously stated, however, testing specimens were all prepared with high relative density (as shown in Table 1), which can introduce shear or expansion bands [17,27]. If only 1D-T data are available, first-order statistics would be expected to show this type of failure at the normalized height of $y_{norm}=0.2$ and at $y_{norm}=0.75$ (Figure 6), which corresponds approximately to the heights of transition of the compaction layers for the vibratory-compaction specimens.

#### 3.3.2. “1D-T” Radial Displacement Field

**Figure 9:**
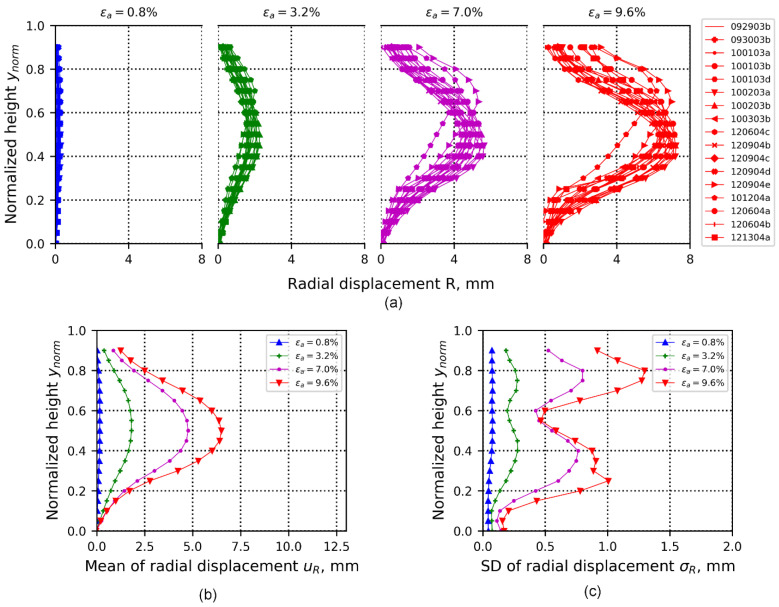
(**a**) 1D-T data ensembles of averaged radial displacements calculated from 17 tests at four loading stages—0.8%, 3.2%, 7.0%, and 9.6% of axial strain. The radial displacement at each specimen height is estimated through averaged radial displacements that are captured by 3D-DIC. (**b**) Mean of data ensembles shown in (**a**). (**c**) Standard deviation of data ensembles shown in (**a**).

The 1D-T averaged radial displacement field presents the sequence of deformation similar to the averaged vertical displacement shown in the previous section. The deformation evolution makes explicit the effect of the friction end at the bottom of the specimen (Figure 9a), indicated by a zero radial displacement induced by the contact between sand and the porous stone [28]. At the top of the specimen, data are incomplete, as mentioned before, because of DIC’s inability to retrieve data close to the boundary of the sand specimen and the porous stone. A visual inspection of the likely extension of the averaged radial displacements shows that some data profiles would not converge to zero, however, which may be associated with likely lateral displacement or tilting of the specimen, induced either by the boundary conditions (seating of top plate) or by local material heterogeneity. The radial displacement at the center of the specimen (around mid-height of the specimen) generally increases, leading to a “bulging” effect. First-order statistics of this displacement field are presented in Figure 9a,b, which capture the progression of the mean and standard deviation of averaged radial displacements, respectively. Progression of the mean (Figure 9b) shows a symmetrical behavior with respect to the mid-height of the specimens, providing a sense of uniformity. Figure 9c, on the other hand, shows significant local variabilities at about $y_{norm}=0.3$ and $y_{norm}=0.80$, which reflects an increase in randomness around the heights where soil layers transition for the vibratory-compaction specimens, suggesting local deformation effects were likely induced by material heterogeneity.

**Figure 10:**
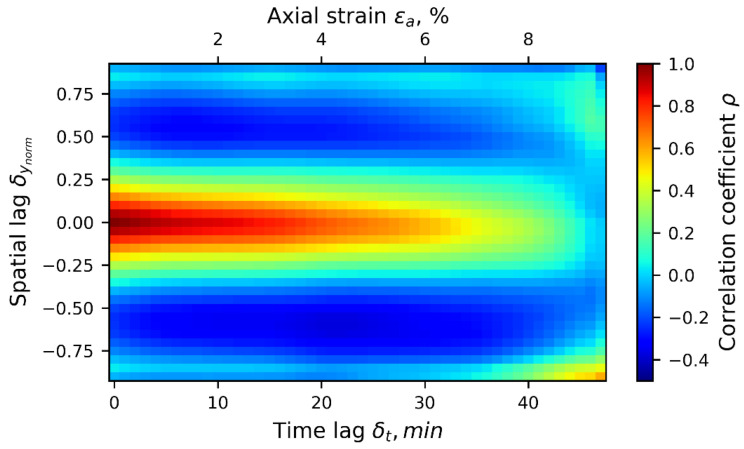
Spatio-temporal empirical correlation map of 1D-T radial data ensemble.

Figure 10 is similar to Figure 8, but is for the second-order statistics of the spatio-temporal correlation or correlation structure of the 1D-T averaged radial displacement data ensemble. Correlation values higher than, for example, ρ>|±0.75| extend to a vertical lag distance of $\delta_{y_{norm}}<\left|\pm 0.20\right|$ and to a time lag of $\delta_{t}<\left|\pm 20\right|$ (time lag equivalent to ±2.0% of axial strain). Beyond this region, the influence of local data drops in space and time, due to the randomness effects associated with the development of local deformation phenomena. In addition, correlation becomes negative for regions at $\delta_{y_{norm}}=\pm \left[0.5,0.75\right]$, indicating generally opposite radial deforming trends for two points at this range of spacing. This can be interpreted as being a trend for any two points spaced at such distance along the specimen’s vertical direction to exhibit opposite radial deformation behaviors.

Similar to the averaged vertical displacement fields, localization effects along the specimen’s boundary surface cannot be fully characterized by interpreting first-order statistics (Figure 9). If only 1D-T data are available, first-order statistics should indicate that this type of failure would show at $y_{norm}=0.3$ and at $y_{norm}=0.80$ (Figure 9), which corresponds approximately to the heights of transition of the compaction layers for the vibratory-compaction specimens.

### 3.4. “3D-T” Data Ensemble

**Figure 11:**
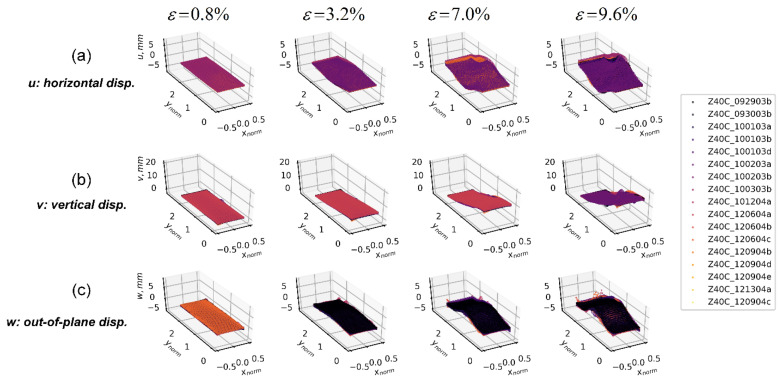
A 3D-T data ensemble (clouds of points) under the Cartesian coordinate system at four loading stages—0.8%, 3.2%, 7.0%, and 9.6% of axial strain. Coordinates are normalized according to the specimen’s diameter. (**a**) Horizontal (u) displacement data ensembles. (**b**) Vertical (v) displacement data ensembles. (**c**) Out-of-plane (w) displacement data ensembles.

**Figure 12:**
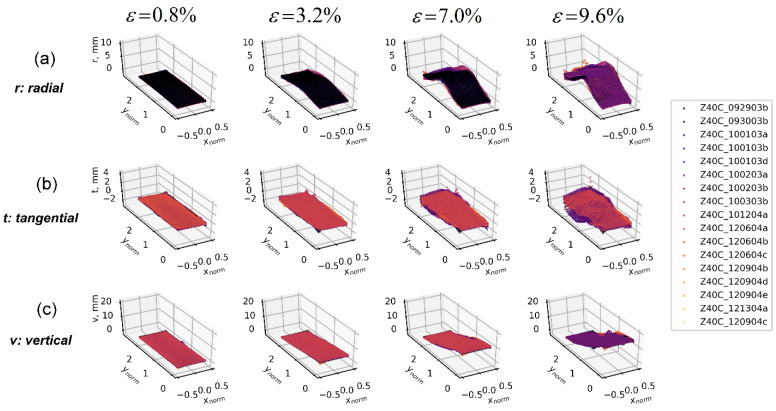
A 3D-T data ensemble (clouds of points) under the cylindrical coordinate system at four loading stages—0.8%, 3.2%, 7.0%, and 9.6% of axial strain. Coordinates are normalized according to the specimen’s diameter. (**a**) Radial (r) displacement data ensembles. (**b**) Tangential (t) displacement data ensembles. (**c**) Axial (v) displacement data ensembles.

The 3D-T data ensemble consists of a set of spatio-temporal displacement fields captured by the 3D-DIC technique over the boundary of sand specimens during the course of triaxial compression. These experimental data include the Cartesian components of hundreds of thousands of displacement vectors on a segment of the cylindrical surface of the specimens [2,23,26]. One single test produces three sequences of clouds of points representing each of the cumulative displacement fields, $u\left(x_{norm},y_{norm}\right)$, $v\left(x_{norm},y_{norm}\right)$, $w\left(x_{norm},y_{norm}\right)$, where $x_{norm}$ and $y_{norm}$ represent the horizontal and vertical normalized coordinates (i.e., material coordinates) with respect to the diameter and the height of the specimen, respectively (each displacement component is projected in a vertical plane formed by the $x_{norm}$ and $y_{norm}$ axis). Since each test produces a different spatial calibration and captures different boundary data coverage areas over each sand specimen, a uniform grid of spatio-temporal points common to the surfaces covered by all tests was defined (common overlapped area at a given space and time), where each displacement field was interpolated on the mesh grid to produce a uniform spatio-temporal data ensemble shared by the three components of the displacement field [2]. This configuration supports the computation of the ensemble’s first- and second-order statistics for each displacement field at the same point in space and time. In addition, this work presents two different projections of the same 3D-DIC data ensemble: one for Cartesian components and one for cylindrical components, as shown in Figure 11 and Figure 12, respectively (clouds of points). One advantage of evaluating displacements under the cylindrical coordinate is that all triaxial samples in this study were constituted into a cylindrical shape; thus, displacements decomposed along radial, tangential, and axial directions can explicitly show how deformation changed the specimen geometry, in contrast with displacements under the Cartesian coordinates, which show the deformation projected onto rectangular planes. Video clips of the deformation sequences of both components’ representations can be found at the Texas Data Repository (https://dataverse.tdl.org/dataverse/SGL-MDPI-Topic-StochasticGeomechancis-ForwardModeling accessed on 9 March 2022) as described in [26].

#### 3.4.1. First-Order Statistics

**Figure 13:**
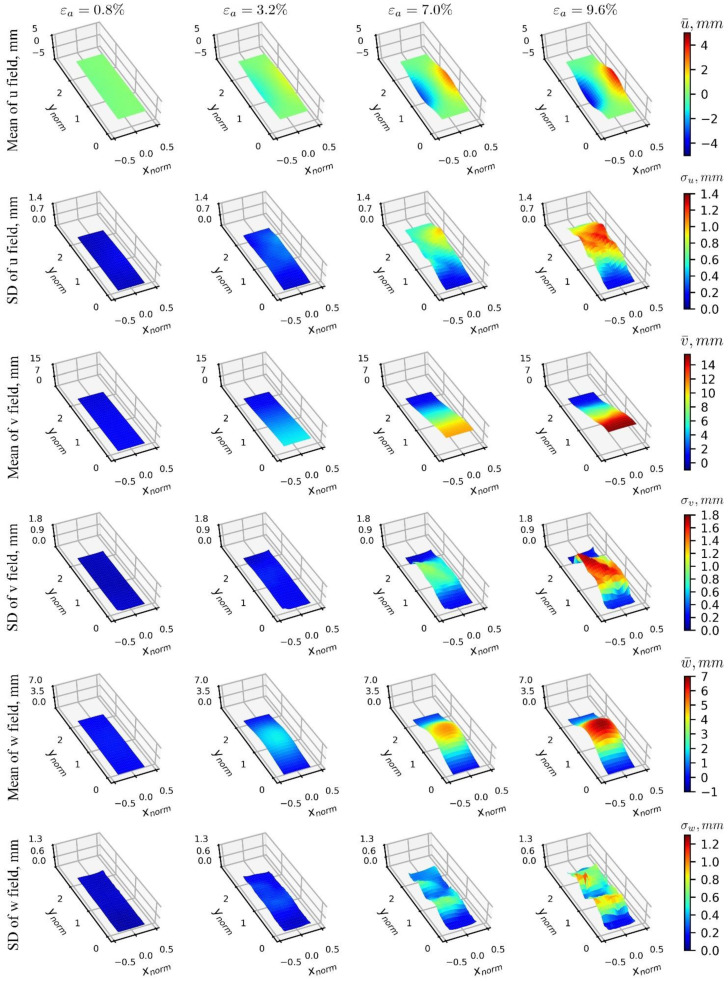
Mean and standard deviation distributions of 3D-T data ensemble under the Cartesian coordinate system, where each column defines a specific loading stage, and each row shows either the mean or standard deviation of a displacement’s data ensemble.

**Figure 14:**
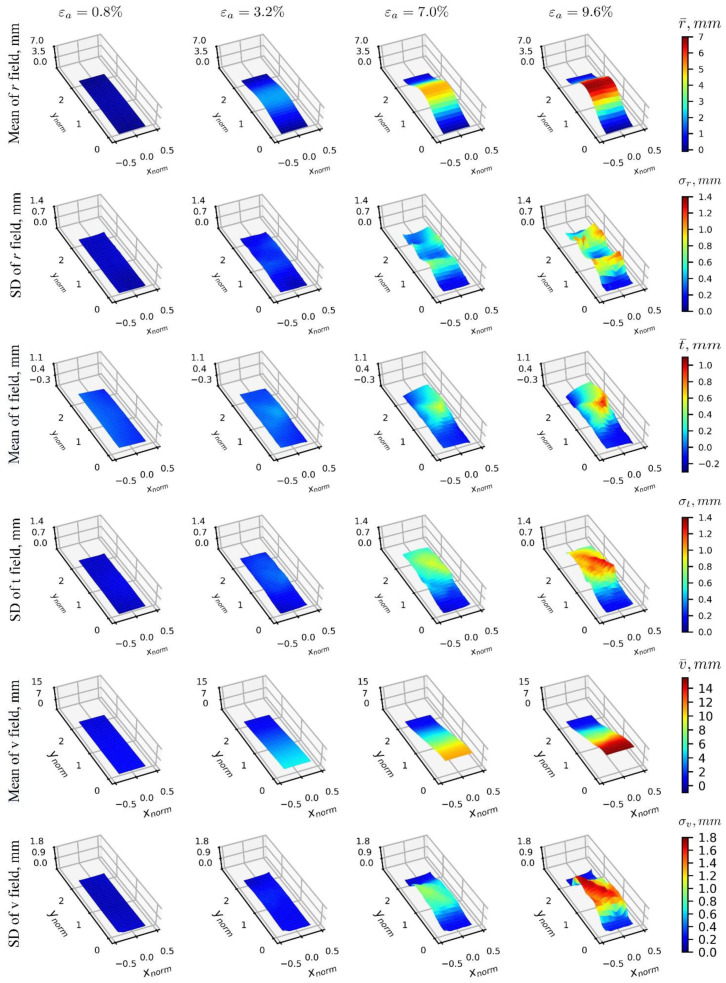
Mean and standard deviation distributions of 3D-T data ensemble under the cylindrical coordinate system, where each column defines a specific loading stage, and each row shows either the mean or standard deviation of a displacement’s data ensemble.

First-order statistics of the 3D-T data ensemble are presented in Figure 13 and Figure 14 for the Cartesian and cylindrical coordinate systems, respectively. Each column in these figures presents a given loading stage, and each row indicates either the mean or standard deviation of a displacement’s data ensemble. Under the Cartesian coordinates, the mean plot of $u\left(x_{norm},y_{norm}\right)$ (horizontal) displacement fields (Figure 13) shows that the specimen is primarily expanded from its vertical center line, and that the material motion is toward two opposite directions. The standard deviation of displacements shows a significant increase starting at $\varepsilon_{a}=7.0\%$, with highest variability in diagonal parallel patterns, indicating the presence of likely competing local deformation effects such as shear bands. Along the vertical direction, the mean surfaces of $v\left(x_{norm},y_{norm}\right)$ displacement fields show a relatively smooth and uniform displacement sequence, consistent with what was depicted in Figure 6 for the 1D-T data ensemble, indicating a uniform displacement from the bottom, from where the specimen is being loaded, and zero vertical displacement at the top. The corresponding standard deviation shows a significant increase starting at $\varepsilon_{a}=7.0\%$ of the axial strain. A unique pattern is evident in the diagonal direction, consistent with that for the $u\left(x_{norm},y_{norm}\right)$ component. Patterns in the $w\left(x_{norm},y_{norm}\right)$ field (out-of-plane direction) highlight the bulging effect, which can be observed at the mid-height of the specimen. The standard deviation shows increasing values at $\varepsilon_{a}=7.0\%$, around the heights where the compaction layers are located, indicating a likely local effect due to variability of the displacement component.

Figure 14 presents results of 3D-T data ensembles under the cylindrical coordinates. The mean field of radial displacement, $r\left(x_{norm},y_{norm}\right)$, is observed at the mid-height of the specimen, and its standard deviation shows peaks around the vertical heights where the transition between the soil layers occurs in the vibratory-compaction specimens, indicating significant variation in the displacement fields in both regions, which can be associated with likely local deformation effects. The tangential displacements field (mean of $t\left(x_{norm},y_{norm}\right)$) indicates that along the off-diagonal direction, soil clusters tend to rotate in a counterclockwise manner (positive value in tangential displacement) that agrees well with previous findings about shear bands’ development [17,27]. The uncertainty of the tangential displacement field is consistent with that of the horizontal displacement field, $u\left(x_{norm},y_{norm}\right)$, observed in the Cartesian coordinate representation (Figure 13), where the predominant pattern of shear band formation seems to be associated with the highest values of standard deviation. The representation of the vertical displacement component, $v\left(x_{norm},y_{norm}\right)$, is the same as the one presented for Cartesian coordinates, indicating a uniform displacement progression by the mean, and that the highest uncertainty values are along the same diagonal, where hypothesized to capture a pattern of shear band formation.

#### 3.4.2. Second-Order Statistics

To calculate the empirical correlation structure of 3D-T displacement fields, each coordinate point, $p_{i}$, is assumed to be a random variable defined in space and time: $p_{i}=\left(x_{norm,i},y_{norm,i},t_{i}\right)$. Therefore, the correlation coefficient between two random variables can be defined as a function of their spatio-temporal lag distance ($\delta_{x_{norm}},\delta_{y_{norm}},\delta_{t}$). The calculation of the empirical covariance, for instance, for the $u\left(x_{norm},y_{norm}\right)$ displacement field, can be defined as:

(5) $${\mathrm{cov}}_{u}(p_{1},p_{2})=E\{[u(p_{1})-\bar{u}(p_{1})][u(p_{2})-\bar{u}(p_{2})]\}=\frac{1}{N}\sum_{i=1}^{N}\left[u_{i}(p_{1})-\bar{u}(p_{1})\right][u_{i}(p_{2})-\bar{u}(p_{2})]$$

where $p_{1}$ and $p_{2}$ represent two coordinates:$\left(x_{norm,1},y_{norm,1},t_{i}\right)$ and ($x_{norm,2},y_{norm,2},t_{i}$), respectively. u represents the displacement data at point $p_{i}$ and $\bar{u}$ represents the data mean at point $p_{i}$. The empirical correlation coefficient is thus calculated as:

(6) $$\rho_{u}(p_{1},p_{2})=\frac{{\mathrm{cov}}_{u}(p_{1},p_{2})}{\sigma_{u}(p_{1})\sigma_{u}(p_{2})}$$

where $\sigma_{u}\left(p_{i}\right)$ means the standard deviation of the displacement data at point $p_{i}$.

**Figure 15:**
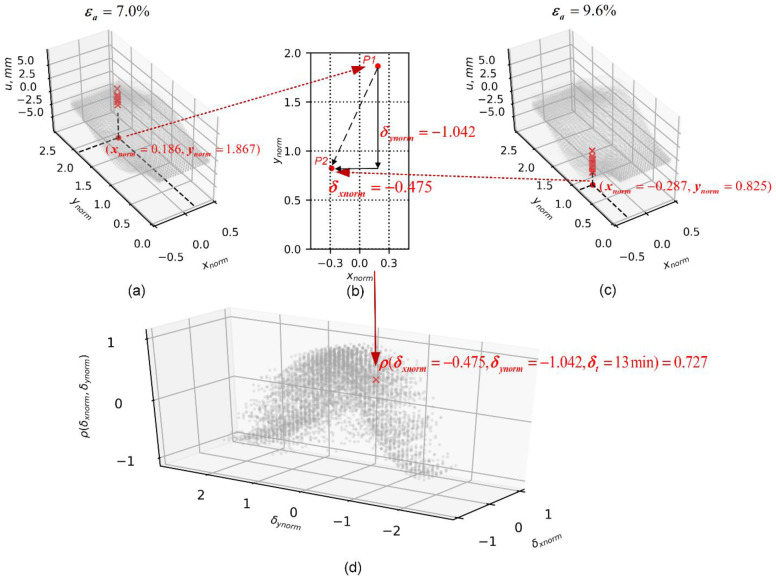
Computation of spatio-temporal correlation coefficients for 3D-T data ensembles. (**a**) Spatial coordinates of first displacement vector, u(P1). (**b**) Spatial lags between u(P1) and u(P2). (**c**) Spatial coordinates of second displacement vector, u(P2). (**d**) Resultant correlation coefficient defined by spatio-temporal lags.

Figure 15 illustrates the process of computing the spatio-temporal correlation coefficient from the 3D-T data ensemble, using as an example the u displacement field at two different deforming stages (7.0 and 9.6%). The floor of the 3D plots represents the plane $\left(x_{norm},y_{norm}\right)$ and the vertical axis represents the *u* component. Figure 15a,c show two displacement vectors, u(P1) and u(P2), at the two deformation levels selected. Each vector consists of 17 data entries that are the result of triaxial compression tests. The spatial lags $\delta_{xnorm}=-0.475$ and $\delta_{ynorm}=-1.042$ can be explicitly presented if we project these two spatial variables on the $\left(x_{norm},y_{norm}\right)$ plane, as shown in Figure 15b. In addition, the time lag can be determined by the difference in axial strain ($\delta_{t}=13min$ or Δε=2.6%), and the calculation of the correlation coefficient will result in a data point in the correlation field, characterized by lags $\delta_{xnorm}=-0.475,\delta_{ynorm}=-1.042$ and $\delta_{t}=13\;\min$. By iterating this process through each pair of displacement vectors, we can obtain the coefficient cloud field, as shown in Figure 15d.

**Figure 16:**
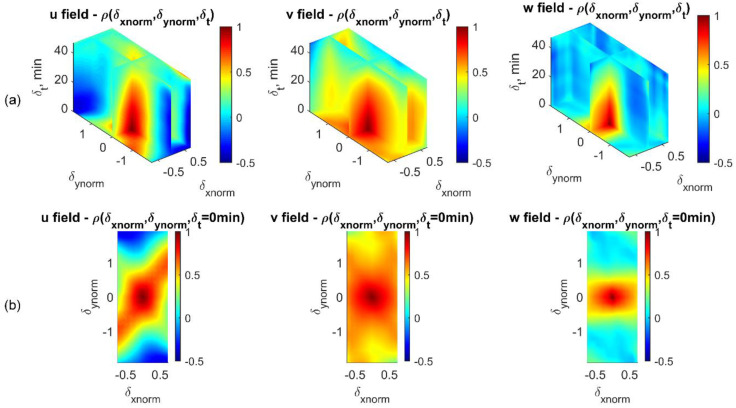
Spatio-temporal empirical correlation structures of 3D-T data ensembles under Cartesian coordinates. (**a**) Smooth representation of correlation structures for *u*, *v*, and *w* displacement fields (left to right). (**b**) Spatial correlation maps for *u*, *v*, and *w* displacement fields when $\delta_{t}=0\;\min$ (i.e., floor of (**a**)).

**Figure 17:**
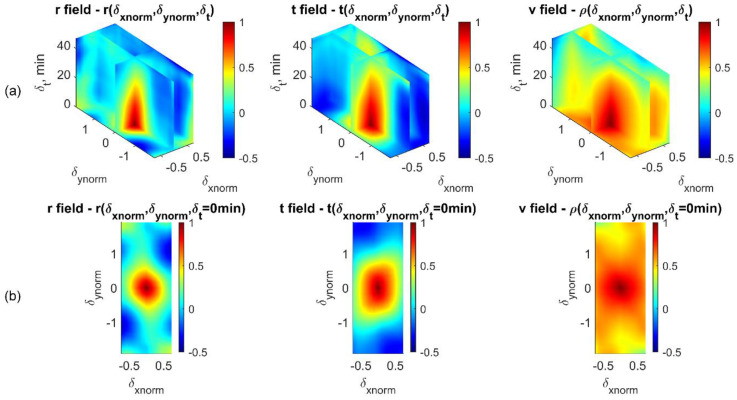
Spatio-temporal empirical correlation structures of 3D-T data ensembles under cylindrical coordinates. (**a**) Smooth representation of correlation structures for *r*, *t*, and *v* displacement fields (left to right). (**b**) Spatial correlation maps for *r*, *t*, and *v* displacement fields when $\delta_{t}=0\;\min$ (i.e., floor of (**a**)).

Once the empirical correlations are calculated for all loading phases (from 0.0 to 9.6% of the axial strain), a hypersurface can then be fitted, using the cubic spline interpolation for the correlation coefficient cloud as an approximate representation of the spatio-temporal correlation structure of each displacement field. The process is similar to that for generating spatio-temporal correlation maps for the 1D-T data ensembles (Figure 8 and Figure 10), except for the additional temporal lag dimension. The resulting 4D volume hypersurface-fitting visualization for all empirical correlation coefficients, with *u*, *v*, and *w* displacement fields, is presented in Figure 16, and that for the *r*, *t*, and *v* fields is presented in Figure 17. These figures show how correlation structures gradually collapse at different rates with increasing spatial and time lags across all displacement fields, and show distinct correlation structure patterns.

To further explain the correlation pattern of the 4D volume shown in Figure 16a and Figure 17a, additional sub-plots (Figure 16b and Figure 17b) showing the floor of the 4D volumes are included that represent auto-correlation maps (i.e., $\delta_{t}=0\;\min$). Within the $u\left(x_{norm},y_{norm}\right)$ displacement field, as shown in the first sub-plot of Figure 16b, two intense correlation bands oriented along diagonal and off-diagonal directions imply shear bands have caused material translational dependencies in the horizontal direction. Along the vertical direction, $v\left(x_{norm},y_{norm}\right)$ displacements show positive correlations for nearly their entire spatial domain (second sub-plot of Figure 16b), meaning that deformation along the vertical direction shows a more predictable spatial pattern, which is consistent with the direction of the deformation and to some degree with the vertical symmetry of the sample compaction. The out-of-plane displacement field, $w\left(x_{norm},y_{norm}\right)$, shows the most significant correlation region at zero spatial lag, suggesting that its predictive domain of influence is shorter than the domain of influence for the other two displacement fields.

The correlation structure presented under the cylindrical coordinate system shows that along the radial direction (the first sub-plot of Figure 17b), negative correlation appears at the normalized heights of −1 and 1 of the specimen. This agrees with our findings in the correlation analysis of 1D-T radius displacements, which shows an opposite radial deforming trend if two local areas are spaced at around half of the specimen height. For correlations of the tangential displacement field, *t* (the second sub-plot of Figure 17b), patterns along diagonal and off-diagonal directions show signs of shear-band formations, but not obviously. A further characterization of displacement gradient and local kinematics [3,29] is anticipated to better present and elucidate the statistics of shear-band properties.

The above results not only offer statistical insights into data ensembles, but the first- and second-order statistics we obtained are essential elements for simulating random displacement fields. If studying the random field satisfies the stationarity and Gaussianity criteria, then these random responses can be reproduced by Gaussian random simulation consistent with the statistics from the experimental observations [23]. Otherwise, some other method such as Polynomial Chaos Expansion (PCE) [23,30] can simulate non-stationary and non-Gaussian random fields. In either case, local deformation phenomena over continuous displacement fields would show infinite virtual simulations which were not sampled but statistically likely consistent with the observed experimental behavior. This work presents all ingredients needed to perform such simulations for the given dimensionality conditions.

## 4. Conclusions

Natural soils possess an inherent variability associated with their engineering and physical properties. In this paper, we introduced first- and second-order statistical analysis performed on boundary displacement observations sampled from a series of nominally similar triaxial compression tests. Results provided insight into the overall deformation modes and inherent uncertainties, as well as spatio-temporal correlation patterns of displacement fields of soil specimens undergoing three-dimensional stress conditions.

First-order statistics of the 0D-0T dimensionality showed the empirical vs. common theoretical distributions of soil’s key parameters, showing, in some cases, deviation from traditional probability models used to simulate its random behavior (i.e., Gaussian and lognormal).

The first-order statistics of boundary 1D-T vertical displacements show that a specimen is mainly deformed with three distinct blocks along the vertical direction—the rigid-body upward motion at the bottom, the linear decrease in vertical displacement in the middle, and the nonlinear decrease in displacement at the top. Furthermore, spatio-temporal correlation analysis reveals significant vertical-displacement correlation in the range of normalized heights between $y_{norm}=0.3$ and at $y_{norm}=0.80$, which can be associated with the development of localization effects such as shear or compaction bands.

The similar analysis performed on 1D-T radial displacement fields showed significant local variability in the normalized specimen heights at $y_{norm}=0.3$ and $y_{norm}=0.80$, which reflects an increase in randomness around the heights where soil layers transition for the vibratory-compaction specimens, reflecting the likely local deformation effects induced by material heterogeneity. In addition, spatial correlation becomes negative for regions at $\delta_{y_{norm}}=\pm \left[0.5,0.75\right]$, indicating a general opposite radial deforming trend for two points at this range of spacing.

Statistics of 3D-T full-field measurements suggest deformation patterns are greatly affected by the variability of localization behavior, such as the development of expansion and shear bands. The presence of shear and expansion bands can also introduce deformation dependencies in space and time.

Results of this work presented the first- and second-order statistics of displacement fields populated from the triaxial sand specimens, which are the essential elements for the stochastic simulation of random fields. In the future work, we will aim at developing the stochastic model capable of simulating “virtual” displacement fields and their corresponding failure mechanisms that are consistent with “real” ones sampled from the laboratory.

## Figures and Tables

**Table 1:** Summary of sample characteristics.

Test Name	Aspect Ratio	Initial Density (kg/m^3^)	Relative Density (%)	Friction Angle (Deg)	Peak $\left(\sigma_{1}^{\prime }/\sigma_{3}^{\prime }\right)$	Sample Preparation
092903b	2.18	1710.95	91.09	49.51	7.35	Vibratory compaction
093003b	2.19	1696.00	85.96	47.98	6.78	Vibratory compaction
100103a	2.21	1702.22	88.10	48.66	7.03	Vibratory compaction
100103b	2.19	1717.13	93.18	47.96	6.77	Vibratory compaction
100103d	2.18	1702.41	88.17	47.37	6.57	Vibratory compaction
100203a	2.20	1715.32	92.57	48.90	7.12	Vibratory compaction
100203b	2.17	1711.91	91.41	47.96	6.77	Vibratory compaction
100303b	2.22	1718.70	93.71	48.56	6.98	Vibratory compaction
120604c	2.25	1717.48	93.30	48.89	7.11	Vibratory compaction
120904b	2.25	1720.40	94.28	48.76	5.86	Vibratory compaction
120904c	2.25	1713.13	91.83	48.77	5.86	Vibratory compaction
120904d	2.24	1707.89	90.04	47.68	5.44	Vibratory compaction
120904e	2.25	1718.70	93.71	47.79	5.51	Vibratory compaction
101204a	2.24	1708.03	90.09	48.03	6.89	Dry pluviation
120604a	2.23	1721.06	94.50	49.46	7.33	Dry pluviation
120604b	2.25	1715.13	92.50	48.54	6.98	Dry pluviation
121304a	2.24	1721.73	94.73	49.30	7.27	Dry pluviation
**First-order statistics of experimental data ensemble**						
Mean	2.22	1712.83	91.72	48.48	6.68	-
Standard deviation	0.03	7.20	2.45	0.62	0.61	-

**Table 2:** First-order statistics of mechanical constitutive parameters.

Statistics	Young’s Modulus (MPa)	Poisson’s Ratio	Friction Angle (Deg)	Dilation Angle (Deg)
Mean	25.70	0.25	43.89	21.23
Standard deviation	5.70	0.16	1.19	2.60
Minimum	20.67	0.07	41.74	12.94
Maximum	40.68	0.49	47.14	24.55

## Data Availability

All data used in this paper, including readings from the triaxial device (global response) and boundary displacement fields (local response) as captured by the 3D-DIC, are available at https://dataverse.tdl.org/dataverse/SGL-MDPI-Topic-StochasticGeomechancis-ForwardModeling accessed on 9 March 2022.
